# Exploring Changes in Land Surface Temperature Possibly Associated with Earthquake: Case of the April 2015 Nepal Mw 7.9 Earthquake

**DOI:** 10.3390/e22040377

**Published:** 2020-03-26

**Authors:** Shunyun Chen, Peixun Liu, Tao Feng, Dong Wang, Zhonghu Jiao, Lichun Chen, Zhengxuan Xu, Guangze Zhang

**Affiliations:** 1 State Key Laboratory of Earthquake Dynamics, Institute of Geology, Beijing 100029, China; liupx@ies.ac.cn (P.L.); jzh@ies.ac.cn (Z.J.); dzsclc@ies.ac.cn (L.C.); 2 China Railway Eryuan Engineering Group Co. Ltd., Chengdu 610031, China; fengtao@ey.crec.cn (T.F.); wangdong13@ey.crec.cn (D.W.); xuzhengx@ey.crec.cn (Z.X.); zhanggz@ey.crec.cn (G.Z.); 3 College of Earth Sciences, Guilin University of Technology, Guilin 541006, China

**Keywords:** Nepal Mw 7.9 earthquake, land surface temperature, the temperature change associated with earthquake, MODIS/Aqua, MODIS/Terra

## Abstract

Satellite thermal infrared remote sensing has received worldwide attention in the exploration for earthquake precursors; however, this method faces great controversy. Obtaining repeatable phenomena related to earthquakes is helpful to reduce this controversy. In this paper, a total of 15 or 17 years of Moderate-resolution Imaging Spectroradiometer (MODIS)/Aqua and MODIS/Terra satellite remote sensing land surface temperature (LST) products is selected to analyze the temperature changes before and after the Mw 7.9 earthquake in Nepal on 25 April 2015 and to explore possible thermal information associated with this earthquake. Major findings are given as follows: (1) from the time course, the temperature slowly cooled before the earthquake, reached a minimum at the time of the earthquake, and returned to normal after the earthquake. Since these changes were initiated before the earthquake, they may even have been precursors to the Nepal earthquake. (2) From the space distribution, the cooling areas correspond to the seismogenic structure during the earthquake. These cooling areas are distributed along the Himalayas and are approximately 1300 km long. The widths of the East and West sides are slightly different, with an average temperature decrease of 5.6 °C. For these cooling areas, the Western section is approximately 90 km wide and 500 km long; the East side is approximately 190 km wide and 800 km long. The Western side of the cooling strips appeared before the earthquake. In short, these kinds of spatial and temporal changes are tectonically related to the earthquake and may have been caused by the tectonic activity associated with the Nepal earthquake. This process began before the earthquake and therefore might even be potentially premonitory information associated with the Nepal earthquake.

## 1. Introduction

For a period of time in the past, it had been strongly claimed by some researchers that earthquakes are unpredictable catastrophes, based on the pervasive hypothesis that the Earth’s crust is in a state of perpetual self-organized criticality in which any small earthquake may cascade into a large event [1,2]. The view of earthquake unpredictability seemed to have swept the world. However, it has been substantiated by observational evidence that this hypothesis is far from the truth, at least in regard to large earthquakes [3,4]. Even a self-organized system like weather forecasting can be well predicted to a certain extent. Because great earthquakes usually cause serious damage to humanity and society, the predictability of damaging earthquakes is widely paid attention to, and actually, a lot of attempts over the world have been made to investigate precursors of deadly earthquakes. 

Among many methods for exploring the premonitory signals before an earthquake, satellite thermal infrared remote sensing has received worldwide attention in the exploration of earthquake precursors [5,6,7]. Many examples of thermal infrared anomalies have been reported [6,8,9,10,11,12,13,14,15]. A variety of anomaly extraction methods and their possible physical mechanisms have also been proposed [9,11,16,17,18,19,20,21,22,23,24]. However, there is still a high degree of controversy regarding satellite thermal infrared anomalies [25,26]. This controversy is, at least partially, is related to the complexity in anomalies of thermal infrared remote sensing. The thermal infrared information received by satellites is mainly thermal radiation from the land surface, which has been modified by the atmosphere, including a large variety of complex meteorological and other non-seismic factors. Eliminating the influence of these factors is very important for understanding these kinds of thermal anomalies [27]. Temperature changes produced by meteorological factors can reach the order of a few or even a dozen degrees Celsius, and the abnormal information related to earthquakes may be completely submerged by meteorological changes. Having appropriate methods and detailed data processing to extract thermal information before an earthquake is necessary. In this paper, taking the April 2015 Nepal Mw 7.9 Earthquake as a case, the following aspects are considered. (1) First, determine the known information as thoroughly as possible, especially non-tectonic factors such as atmospheric and solar influences, and then look for possible earthquake-related information from the remaining information. (2) If a piece of information is related to an earthquake, it should be spatially related to the seismogenic structure; it should temporally include coseismic information; and even if there is an earthquake precursor, the change should become more significant at the occurrence of the earthquake. (3) The earthquake-related phenomenon should be repeated when using data from different satellites. The processing of satellite data can also generate uncertainty, especially for long-term satellite data. Using different satellite data for verification is best. If different satellites detect the same phenomenon, then at least the phenomenon itself is reliable. 

A strong earthquake shook Nepal on 25 April 2015, with a moment magnitude of Mw 7.9 and an initial rupture depth of approximately 12–15 km [28]. The epicenter of the mainshock was at 84.708 °E, 28.147 °N and occurred in Gorkha district of Nepal [29]. The earthquake caused severe damage to life and property and was perhaps the worst natural hazard to strike Nepal since the 1934 quake [30]. The Mw 7.9 earthquake in Nepal occurred on the Qinghai-Tibet Plateau, where the mountains are steep, the terrain is complex, and in situ field observations face many difficulties. Satellite remote sensing can provide information on the evolution of changes in time and space. Discovering some earthquake-related clues is possible, which would be valuable for increasing the understanding of the Nepal earthquake [25,30,31] [26]. In this paper, following the above-mentioned three points, a total of 15 or 17 years of both MODIS/Aqua and MODIS/Terra satellite remote sensing LST products from 2002 or 2000 to 2016 are selected to investigate the temperature changes before and after the Mw 7.9 earthquake in Nepal on 25 April 2015 and to explore the thermal anomalies that may have been associated with the earthquake.

## 2. The Geological Background and Research Areas

### 2.1. Geological Background of Southern Margin of the Qinghai-Tibet Plateau

**Figure 1:**
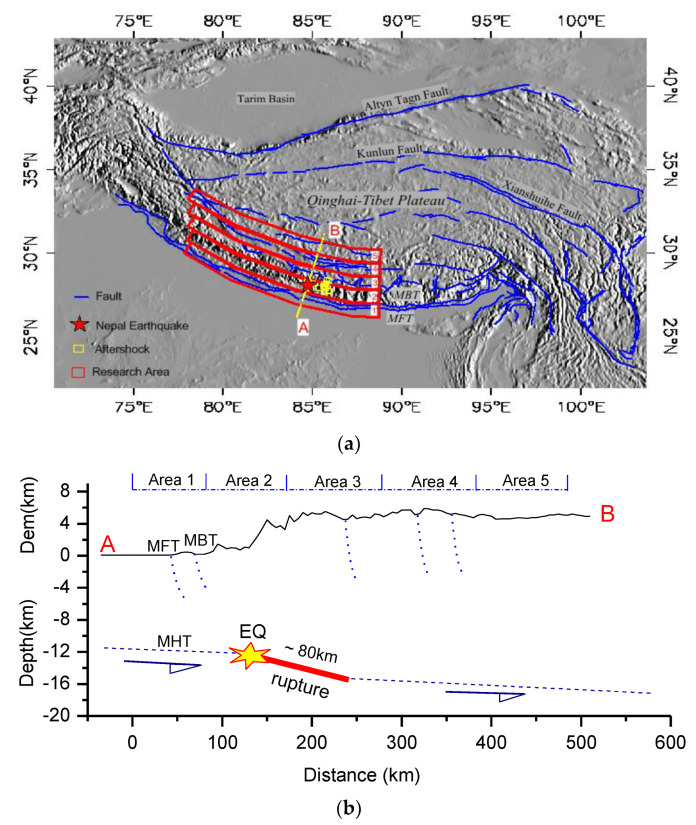
Schematic map of the fault system of the Qinghai-Tibet Plateau and diagrams of the research areas. (**a**) Spatial partition, where numbers 1, 2,..., 5 represent different areas, and AB is the profile position. (**b**) The topographic profile along AB and research areas (Area 1, Area 2,..., Area 5); the seismic rupture is obtained from Reference [36].

Since the Eocene, the Indian Plate and the Eurasian Plate have collided along a plate boundary of approximately 2500 km to form the Himalayan orogenic belt. The cumulative crustal shortening is approximately 2000–3000 km [32]. The collision between the plates produced some thrust faults, like the main boundary thrust (MBT), parallel to the Himalayan arc on the Southern side of the Himalayas (Figure 1a). The main boundary thrust, which constitutes the boundary between the Low Himalaya and the sub-Himalaya, consists of a series of thrust faults with Northern dips. In some locations, the MBT causes the pre-Tertiary strata to thrust. Above the Quaternary strata [33], the Southernmost fault is the main frontier thrust (MFT), which constitutes the boundary between the Indian Plate and the Qinghai-Tibet block and can be seen on the surface, causing the Cirqueke Formation of the New World to be thrust onto the Quaternary sediments of the Ganges Plain [34]. These nearly parallel, North-dipping thrust faults on the surface merge downward into a unified detachment fault, the main Himalayan thrust (MHT) [35]. Many large earthquakes occurred in these thrust faults in the Southern margin of the Qinghai-Tibet Plateau [29].

On 25 April 2015, the Mw 7.9 earthquake in Nepal occurred in the Southern foothills of the Himalayas. The epicenter of the mainshock was at 84.708 °E, 28.147 °N, and the initial rupture depth was approximately 12–15 km [28]. The aftershocks (Mw≥5) that occurred on 25 April 2015 were given in Figure 1a. The focal mechanism solution indicates that the earthquake was a low-angle thrust-type earthquake [29]. Field investigations have not found that the Mw 7.9 earthquake in Nepal formed a surface rupture zone on the main fault in the Southern foothills of the Himalayas. The tectonic nature of the Southern foothills of the Himalayas is a thin-skin structure, which is characterized by the steepening slope of the shallow fault (approximately 7°) and the deep fault (approximately 11°). 

According to the seismic wave inversion results [36], the coseismic rupture extends ~80 km toward the North (Figure 1b) and ~100 km toward the Southeast. Regarding the coseismic stress drop for the 2015 Nepal Mw 7.9 earthquake, the values given by different scholars are quite different. Some researchers have reported that the stress drops were approximately 3.0–3.2 MPa [37]; however, other researchers assume a circular crack model and their spectral characteristics to estimate stress drops of 22.7 ± 1.9 MPa for the mainshock [38]. These values for the stress drops are highly dependent on the model [37]. 

### 2.2. Areas of Research

Considering that the main fault zones in the vicinity of the epicenter (Figure 1a) are nearly parallel and mainly distributed along the front of the Himalayas, this paper selects five parallel research areas and analyzes the statistical characteristics of their time processes. In fact, in the analysis of this paper, the time course of each pixel is processed, and finally, the spatial-temporal evolutionary process of the surface temperature is obtained. Here, the selection of the study area mainly aims at the time process analysis of thermal information.

In addition, the range of the study area has an impact on the regional average value, which influences the magnitude of the anomalous changes. The larger the area is, the smaller the amplitude of the anomaly; the smaller the area is, the larger the amplitude of the anomaly. However, the selection of the size of the study area should not affect the changing trend of abnormality. In addition, if this abnormality actually exists, the same results should be obtained from different satellite data. In this paper, two completely independent observations of satellite data are used for comparative studies to verify the reliability of the thermal anomaly information.

## 3. Data and Methods

### 3.1. Data

The information received by the satellites is mainly surface thermal radiation that has been modified by the atmosphere. There are many uncertainties in the direct use of satellite imagery to analyze crustal activity. Atmospheric correction is one of the basic tasks. Land surface temperature, which contributes to surface thermal radiation, can be obtained from satellite remote sensing thermal infrared data after processing, including the atmospheric correction. The data used in this paper are well-known international LST products [39,40,41]. Therefore, specifically considering atmospheric correction technical issues involved in processing the raw satellite image is not necessary.

The LST products selected in this paper are data from the V5 version of the MODIS/Terra and MODIS/Aqua satellites covering March 2000 or July 2002 to February 2016, which are obtained according to the day/night surface temperature inversion algorithm (Wan et al., 2002; Wan, 2008). These products have a spatial resolution of 0.05 degrees, a time resolution of 8 days, and an accuracy of 1.0 K [40].

**Figure 2:**
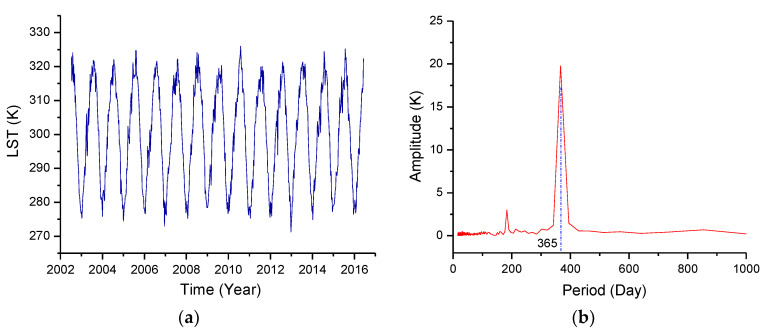
Variations in land surface temperature (LST) with time (**a**) and its spectrum distribution (**b**). Latitude and longitude: 93.7° E, 37.9° N.

Figure 2 shows the variation in LST with time and its spectral distribution at a specific location (latitude and longitude: 93.7° E, 37.9° N). Figure 2 intuitively shows that the largest change in LST is the annual variation. The annual component represents the influence of solar radiation, and the temperature changes at other frequencies are much weaker than the annual variation. Removing the annual variation is a prerequisite for further identifying abnormal information.

### 3.2. Methods

The change in LST is mainly driven by periodic solar radiation, which provides a periodic heat flux. For the Earth, the Sun is a stable periodic heat source, mainly causing daily and annual changes. The solar-synchronous polar-orbiting satellites, such as Terra and Aqua, pass over the same place only twice in one day, and the daily cycle changes are not observed. 

In simple terms, there are the following terms:

(1) $$T\left(t,x,y\right)=T_{0}\left(x,y\right)+T_{a}\left(t,x,y\right)+\Delta T\left(t,x,y\right)$$

where T(t,x,y) represents the LST with unit of K or ℃ , t is time and x and y are spatial coordinates; $T_{0}\left(x,y\right)$ is the stable component of the LST, mainly including the influence of terrain and latitude changes; $T_{a}\left(t,x,y\right)$ is a stable annual component, representing the influence of solar radiation; and ΔT(t,x,y) is the residual part, mainly affected by various factors such as atmosphere, vegetation and tectonic activity. 

Among these terms, $T_{0}\left(x,y\right)$ and $T_{a}\left(t,x,y\right)$ are relatively stable and can be considered together. $T_{sta}\left(t,x,y\right)=T_{0}\left(x,y\right)+T_{a}\left(t,x,y\right)$. $T_{sta}\left(t,x,y\right)$ represents the strongest component of the LST. Additionally, $T_{sta}\left(t,x,y\right)$ is independent of tectonic activity such as earthquakes. Thus, earthquake anomaly information should be included in ΔT(t,x,y). For convenience, ΔT(t,x,y) is called the annual variation residual.

The annual variation residual ΔT(t,x,y) is selected as the object for further analysis:

(2) $$\Delta T\left(t,x,y\right)=T\left(t,x,y\right)-T_{sta}\left(t,x,y\right)$$

In fact, the annual variation residual ΔT(t,x,y) is caused by local factors such as crustal activity, human activities and vegetation, and is often disturbed by thermal information from external atmospheric flows. In the above sense, the annual variation residual ΔT(t,x,y) can be expressed as:

(3) $$\Delta T\left(t,x,y\right)=T_{human}\left(t,x,y\right)+T_{local}\left(t,x,y\right)+T_{atmosphere}\left(t,x,y\right)$$

Among these terms, $T_{human}\left(t,x,y\right)$ represents the temperature change caused by human activities; $T_{local}\left(t,x,y\right)$ represents the temperature change caused by local factors and is called the local temperature; and $T_{atmosphere}\left(t,x,y\right)$ is the temperature change caused by external heat carried by the atmospheric circulation. $T_{human}\left(t,x,y\right)$ and $T_{atmosphere}\left(t,x,y\right)$ are independent of earthquakes, and earthquake anomaly information should be included in $T_{local}\left(t,x,y\right)$.

Now, Equation (3) can be written as:

(4) $$T_{local}\left(t,x,y\right)=\Delta T\left(t,x,y\right)-T_{human}\left(t,x,y\right)-T_{atmosphere}\left(t,x,y\right)$$

In other words, obtaining thermal information related to crustal activity (such as earthquakes) is possible only by extracting the local temperature $\;T_{local}\left(t,x,y\right)$. In the short term, vegetation and other factors do not change significantly. Therefore, the key is to remove the impact of atmospheric circulation on  ΔT(t,x,y).

From the spatial scale, there are obvious differences in the influence of atmospheric circulation and local factors on LST: the spatial scale of the atmospheric circulation is relatively larger, and the spatial scales of local factors are relatively smaller, especially the linear characteristics of the fault zone, which has obvious one-dimensional features. Thus, Equation (3) can be solved by a two-dimensional spatial analysis method, such as wavelet analysis. Then, $T_{local}\left(t,x,y\right)$ can be obtained by Equation (4). If there is thermal information associated with earthquakes, such information should be found in $\;T_{local}\left(t,x,y\right)$. 

### 3.3. Procedure of Data Processing

**Figure 3:**
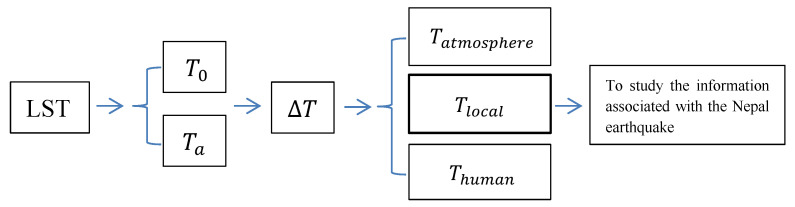
Diagram for the procedure of data processing.

Data processing is roughly divided into two categories: time-based and space-based processes. First, for each location  (x,y), the surface temperature  T(t,x,y) time history is processed to obtain  ΔT(t,x,y). Then, for each time  (t), the spatial information regarding ΔT(t,x,y) is processed to obtain $\;T_{atmosphere}\left(t,x,y\right)$, $T_{local}\left(t,x,y\right)\;$ and $\;T_{human}\left(t,x,y\right)$. Finally, based on $\;T_{local}\left(t,x,y\right)$, the analysis of the thermal anomaly possibly associated with the Nepal earthquake is carried out. The procedure of data processing is shown in Figure 3.

The basic data processing flow is briefly described as follows:

(1) Determine $\;T_{0}\left(x,y\right)$. $T_{0}\left(x,y\right)$ is independent of time and may be obtained through the multiyear average value at location (x,y);

(2) Obtain $T_{a}\left(t,x,y\right)$ and $\;T_{sta}\left(t,x,y\right)$. $T_{a}\left(t,x,y\right)$ is different in different locations, but its time course is determined. For a given location, this value can be obtained from years of data. The specific process includes (i) removing the effects of non-annual components. This removal requires a certain filtering technique to extract the annual variation while filtering out the non-annual components. In this paper, in order to eliminate the influence of non-annual components and the errors introduced by filtering, the one-dimensional orthogonal wavelet theory (Appendix A) is used to extract the annual period components. The advantage of using orthogonal wavelet decomposition is that the extracted annual periodic components do not contain interference by other periodic components [42,43]. (ii) $T_{a}\left(t,x,y\right)\;$ can be obtained by averaging the surface temperatures on the same date from the multiyear data. (iii) According to $T_{0}\left(x,y\right)$ and $T_{a}\left(t,x,y\right)$, $T_{sta}\left(t,x,y\right)\;$ is further obtained (Appendix C).

(3) Calculate ΔT(t,x,y). ΔT(t,x,y) is obtained according to Equation (2), based on $T_{sta}\left(t,x,y\right)$.

(4) Obtain $\;T_{local}\left(t,x,y\right)$. For a fixed area, the satellite imaging time is short; that is, for the same image, the time in each pixel can be considered constant. At the same time, considering that the spatial scale of the atmospheric circulation is usually large, the spatial decomposition of ΔT(t,x,y) at each time can be accomplished to obtain $\;T_{atmosphere}\left(t,x,y\right)$, $T_{local}\left(t,x,y\right)$ and $\;T_{human}\left(t,x,y\right)$. In this paper, the two-dimensional orthogonal wavelet method (Appendix B) is used to spatially decompose  ΔT(t,x,y). ΔT(t,x,y) is decomposed into three spatial scales: less than 80 km × 80 km, greater than 600 km × 600 km, and intermediate-scale components. Among these components, those with spatial scales greater than 600 km are mainly caused by atmospheric circulation, namely, $\;T_{atmosphere}\left(t,x,y\right)$; the changes caused by human activities are concentrated in spatial components of less than 80 km, namely, $\;T_{human}\left(t,x,y\right)$. The remaining components represent the contribution of local factors to the LST, namely, $T_{local}\left(t,x,y\right)$.

To avoid the border effect in the spatial decomposition, the spatial scales involved in the above four steps of data processing are larger than those in Figure 2a, as described in Appendix D.

(5) Calculate the variations in thermal information with time, based on $\;T_{local}\left(t,x,y\right)$. (i) First, according to the study areas (Figure 1), the spatial average results of each study area are separately calculated at each time, so that the time courses of different study areas are obtained. (ii) Then, the statistical parameters, such as the standard mean square error and the histogram of the time process, are calculated separately. Thus, the statistical characteristics of thermal anomaly information that may be related to earthquakes are analyzed further.

## 4. Results

### 4.1. Temporal Process

According to the above procedure, the LST data from the MODIS/Terra and MODIS/Aqua satellites are processed separately. Here, the MODIS/Aqua results are taken as an example for analysis. The MODIS/Terra results are given in the discussion section. In addition, considering that $\;T_{0}\left(x,y\right)$,$T_{a}\left(t,x,y\right)$,$\;T_{human}\left(t,x,y\right)$ and $\;T_{atmosphere}\left(t,x,y\right)$ are independent of earthquake anomalies, these values are not analyzed (see Appendix D). Here, we focus on the local temperature $\;T_{local}\left(t,x,y\right)$.

(A) Analysis of standard deviation

**Figure 4:**
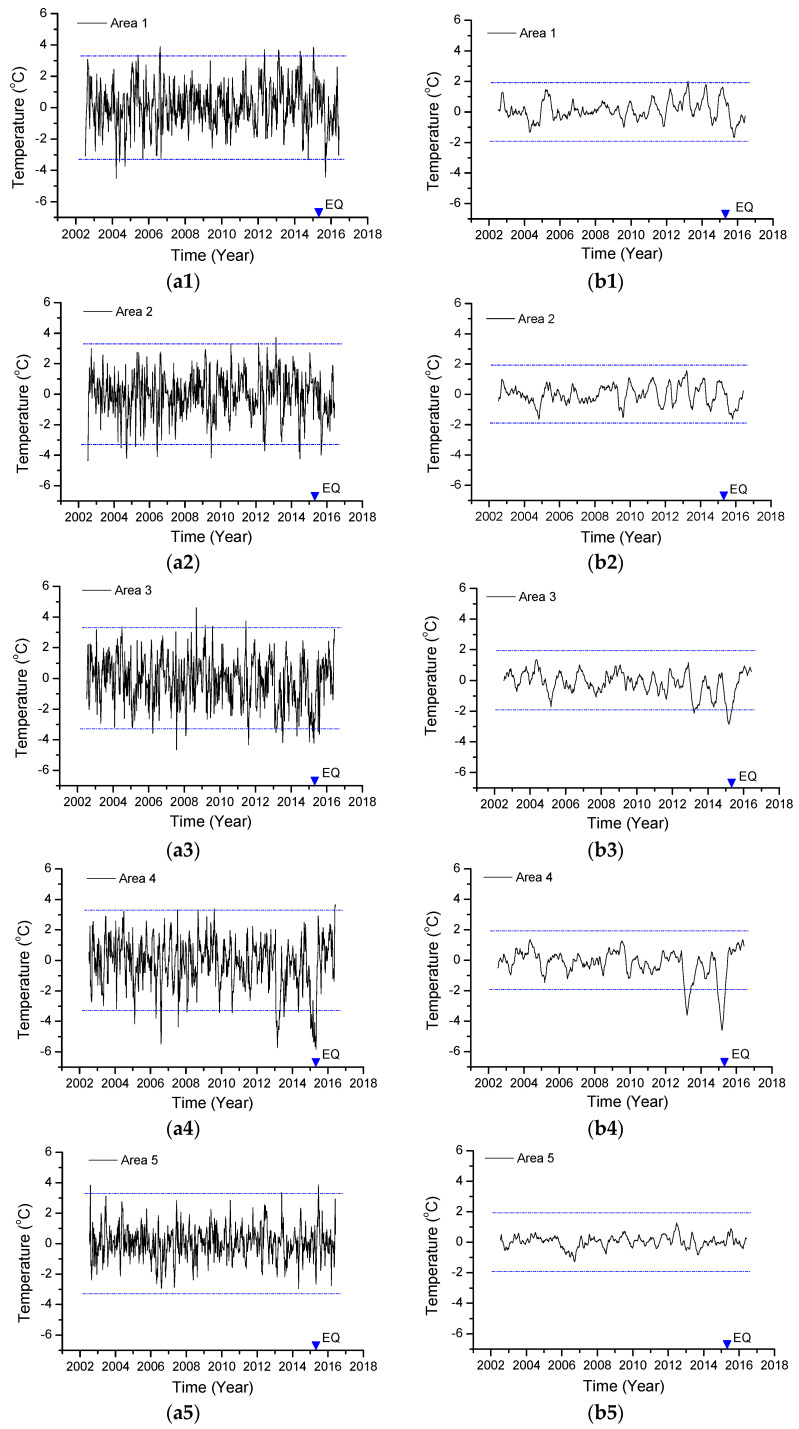
Variations in $T_{local}\left(t,x,y\right)\;$ with time in different study areas. (**a1**–**a5**) are the original $\;T_{local}\left(t,x,y\right)$ values in different study areas; (**b1**–**b5**) are the moving averages (the window length is 112 days). The blue lines represent the double value of the maximum standard deviation among study areas.

Figure 4 shows the variations in the average local temperature $T_{local}\left(t,x,y\right)$ with time in different study areas. The corresponding statistics for these areas are shown in Table 1. Table 1 and Figure 4 show that the temperature fluctuations are between −5.83 and 4.61 °C, the fluctuation ranges of the study areas are approximately equivalent, and the fluctuation range is approximately 8~9 °C; the standard deviation lies between 1.39 and 1.65 °C. Intuitively, there is no obvious increase and decrease. To improve the credibility of the anomaly information, the maximum standard deviation of the five study areas is taken as the reference value, denoted by RefStd. Here, RefStd is 1.65 °C. The double RefStd is 3.30 °C and is shown as a line in Figure 4a1–a5.

As shown in Figure 4a1–a5, there is a phenomenon in which the temperature changes more than the double RefStd during the Nepal earthquake. However, many times before and after the earthquake, there are occasions when the local temperature value exceeds the double RefStd. Sometimes, the local temperature is higher than the double RefStd, and sometimes, local temperature is lower than the double RefStd. Hence, there is not a unique case in which the temperature changes during the earthquake are more than the double RefStd, which means that the thermal anomaly related to the earthquake simply based on the original local temperature value exceeding the double RefStd is insufficient.

(B) Analyses of moving average

Further, as shown in Figure 4a1–a5, the cases when these temperature values exceed the double RefStd are mostly short-term changes. Considering that the meteorological factors are extremely complicated, these factors are usually short-term temperature changes of a few or even a dozen °C per day. Information related to earthquakes can easily be submerged in meteorological changes. To suppress the influence of short-term changes and to highlight the earthquake-related information, the temperature change signals of the above different study areas are smoothed over a long time. In this paper, taking the 112-day moving average window weakens the impact of short-term changes. The results are shown in Figure 4b1–b5, and the relevant statistical information is given in Table 1.

After smoothing, the temperature fluctuation is between –4.6 and 2.02 °C; the standard deviation is significantly reduced to between 0.37 and 0.95 °C. Similarly, the maximum standard deviation of the five areas is 0.95 °C, which is taken as the reference value, denoted as SRefStd. The lines in Figure 4b1–b5 represent the double SRefStd. As shown in Figure 4b1–b5, only study areas 3 and 4 are more than the double SRefStd (Figure 4b3,b4), and both were cooling before the earthquake. Among these areas, the strongest deviation corresponds to the occurrence of the earthquake.

In short, from the time process, there was significant cooling before the earthquake, and the temperature reached the lowest value during the earthquake and returned to normal after the earthquake. The variation range was more than twice the SRefStd, and the change in area 4 reached −4.60 °C, even more than 4 times the SRefStd. That is, in study areas 3 and 4, a change in temperature more than the normal background occurred before the earthquake.

(C) Frequency distribution

According to the previous analysis, the abnormal change in area 4 is the most obvious (Figure 4b4), while the adjacent study area 5 has no obvious abnormality (Figure 4b5). Here, study area 4 and area 5 are selected for further statistical analysis of the frequency distribution to compare the similarities and differences in temperature distributions with and without abnormalities.

**Figure 5:**
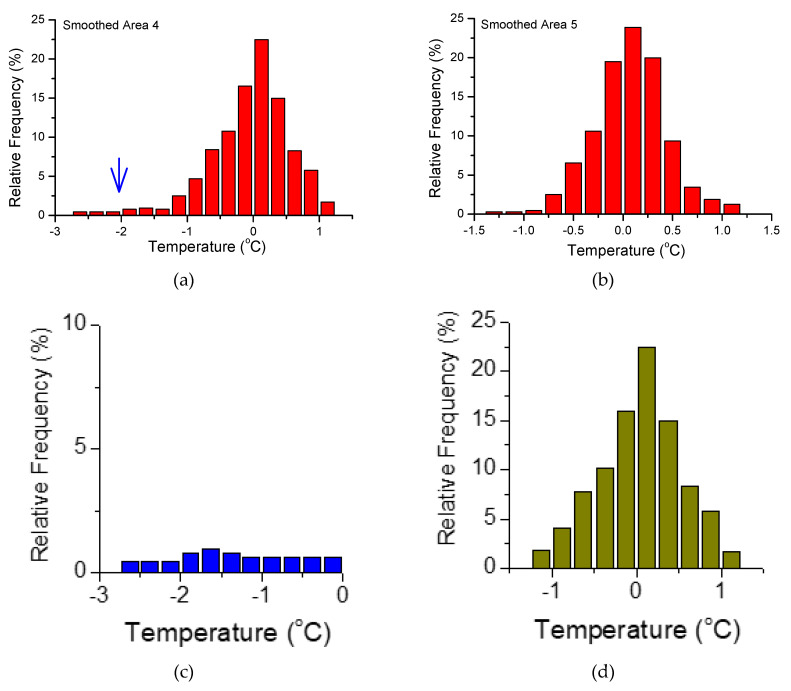
The frequency distribution of the smoothed $\;T_{local}\;$ in study area 4 (**a**) and area 5 (**b**). The distribution in (**a**) can be simply decomposed into the two distributions in (**c**) and (**d**).

Figure 4b and Figure 5a show the frequency distribution of the smoothed temperature values of study area 4 and area 5 (Figure 4b4,b5). Figure 5 illustrates that (i) in the range of [−1.5 ℃, 1.5 ℃], the distributions of the two study areas are similar and should be close to the same distribution pattern (Figure 5a,b); (ii) in the range of [−3.0 ℃, −1.5 ℃], there is another temperature distribution pattern for study area 4, which is close to a uniform distribution (Figure 5c). That is, the temperature distribution of study area 4 includes a temperature distribution pattern different from that of study area 5. We simply decompose the temperature distribution contained in study area 4 into two cases, close to the average distribution and the normal distribution, as shown in Figure 5c,d. Given these two distributions, for the distribution shown in Figure 5c, the value in [−3.0 ℃, −1.5 ℃] is the same as that shown in Figure 5a, and the value in [−1.5 ℃, 0 ℃] is the mean of [−3.0 ℃, −1.5 ℃]. The distribution pattern in Figure 5d is similar to that in Figure 5b. The distribution shown in Figure 5c reflects the temperature distribution of the difference between study areas 4 and 5, and the difference is mainly reflected in the low-temperature part. This result is consistent with the characteristics of the cooling for the time course performance.

### 4.2. Spatial Distribution

**Figure 6:**
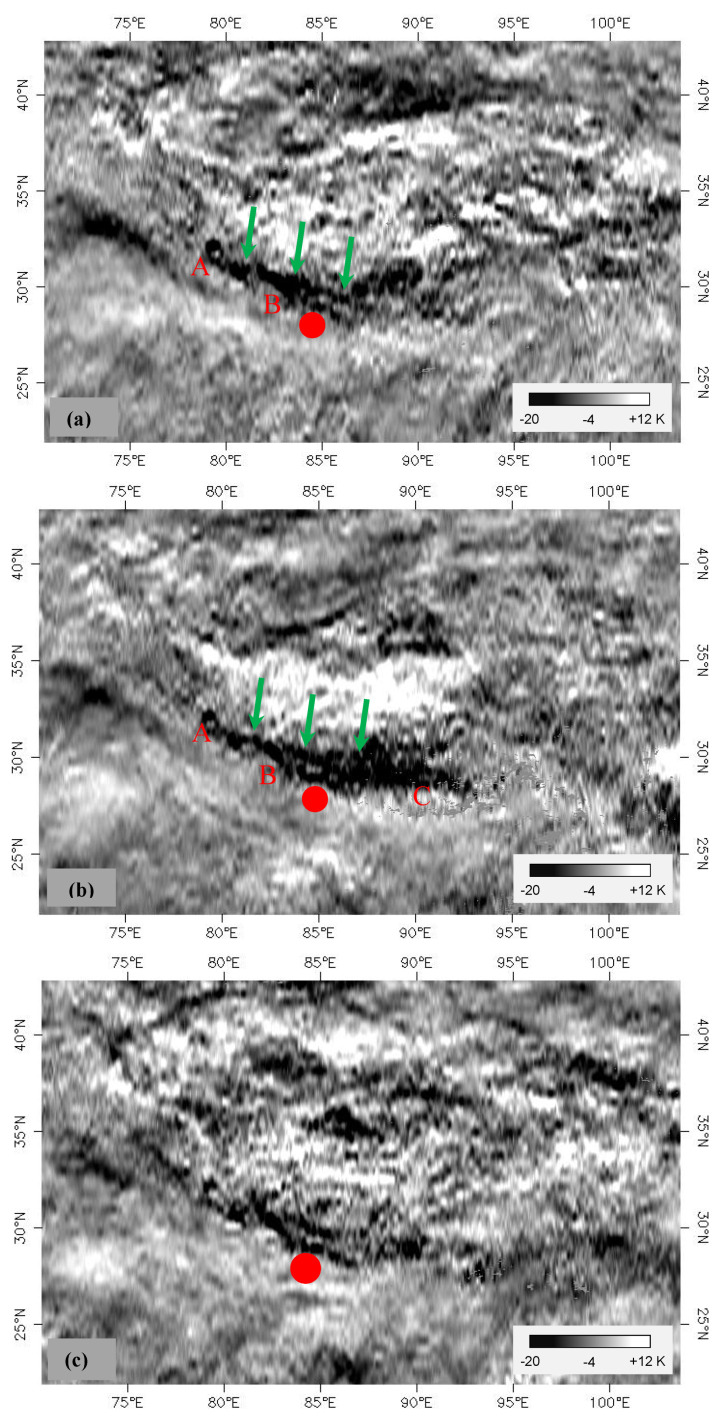
Spatial distributions of local temperature $T_{local}\;$ before and after the Nepal earthquake (Aqua). (**a**–**c**) are pre-earthquake (15–22 April 2015), coseismic (23–30 April 2015) and postearthquake (31 April–7 May 2015), respectively. The red solid cycle is the Nepal earthquake.

Figure 6 shows the spatial distribution of the local temperature $T_{local}\left(t,x,y\right)\;$ before and after the Nepal earthquake. In Figure 6, the most obvious feature is the apparent temperature decrease near the epicenter at the time of the earthquake (Figure 6b), referred to as coseismic cooling. The coseismic cooling is mainly distributed in study area 3 and area 4. After the earthquake, the cooling strip is obviously weakened. The coseismic cooling strips are distributed along the Himalayas and are approximately 1300 km long. The widths of the East and West sides of the coseismic cooling strips are slightly different, with an average temperature decrease of 5.6 ℃. The Western section (AB) is approximately 90 km wide and 500 km long, and the East side (BC) is approximately 190 km wide and 800 km long.

In comparing the spatial distributions of local temperature $T_{local}\left(t,x,y\right)\;$ before and during the earthquake, the abovementioned cooling strip (AB section) appeared on the West side of the earthquake before the earthquake occurred (Figure 6a). That is, the evolution of the cooling strip was from West to East, which is consistent with the extension of the coseismic rupture from West to East [29].

Further, there is still another important phenomenon: from the perspective of seismic location, the epicenter of the Nepal earthquake is approximately 40 kilometers away from the cooling strip (Figure 6b). In fact, the earthquake rupture has a certain length, and the epicenter refers to the rupture starting point. According to the seismic wave inversion results, the coseismic rupture extends ~80 km to the North and ~100 km Southeast [36]. Since the earthquake occurred on a low-angle thrust fault (~15°), the distance extended to depth is projected to the North, which is equivalent to a Northward extension of ~80 km. That is, the stopping boundary of the rupture is already in the cooling strip. In fact, as shown in Figure 1a, some of the aftershocks that occurred on 25 April 2015 were located in the study area 3, namely in the cooling strip. Therefore, although the earthquake epicenter is outside the cooling zone, there is a spatial correlation between the cooling zone and the coseismic rupture during the Nepal earthquake.

In summary, a significant cooling zone is found in the local temperature field $\;T_{local}\left(t,x,y\right)$. Moreover, the coseismic cooling zone is associated with the coseismic rupture distribution, and the evolution direction is highly consistent with that of coseismic rupture. This feature implies that the cooling zone might be related to the evolutionary process of crustal stress.

## 5. Discussion

### 5.1. Comparison of Two Cooling Events before the Earthquake

**Figure 7:**
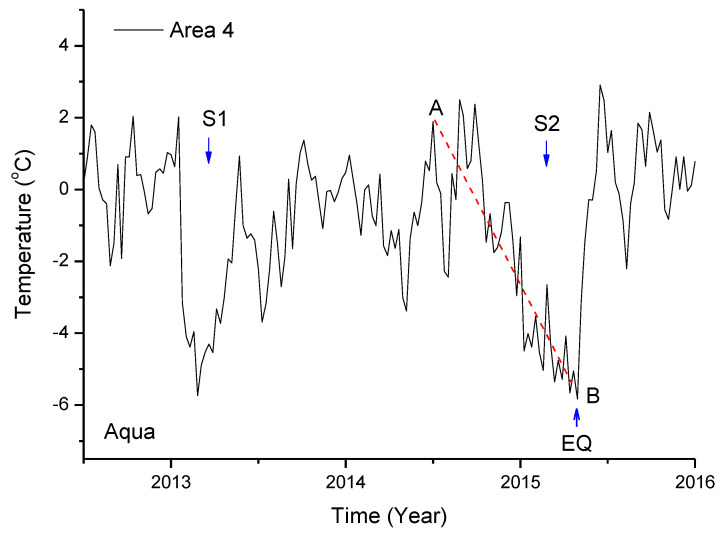
Two cooling events in local temperature $T_{local}$ in study area 4 before the Nepal earthquake (Aqua satellite).

As shown in Figure 4b4, two significant cooling events occurred during the time course in study area 4, which are further analyzed here. For convenience, they are labeled S1 and S2, as shown in Figure 7. S1 was a relatively sharp decrease in temperature. From the normal background to the valley point, S1 experienced approximately 40 days in which the temperature decrease was ~6 °C; then, the temperature increased, and the recovery duration to normal level was close to the decrease duration. During these days, the stage of fastest decrease lasted approximately 16 days. Meanwhile, S2 showed a slow decrease. From the normal background to the valley point, S2 lasted 320 days, and the temperature decrease was ~7 °C. S2 reached the minimum value at the occurrence of the earthquake and recovered to the normal background value soon after the Nepal earthquake.

Although both S1 and S2 are cooling events, there is a significant difference in duration from the normal background to the minimum point. The duration of S1 lies within the range of the duration of weather events. The magnitude of S1 is more than twice the standard deviation (Figure 4a4 or Figure 4b4); however, confirming that S1 was uniquely generated by crustal activity is hard, as its duration is short and might be easily mixed by the effect of weather events (i.e., continuous rainfall or snow).

As for S2, the temperature decrease lasted for nearly a year, and can hardly be interpreted as an event associated with meteorological changes. Particularly, the temperature reached a minimum value when the earthquake occurred and quickly increased back to normal background after the earthquake. This trend of change largely coincided with the occurrence of the earthquake, which implies that the change was related to the earthquake.

### 5.2. Comparison of Data from Different Satellites

**Figure 8:**
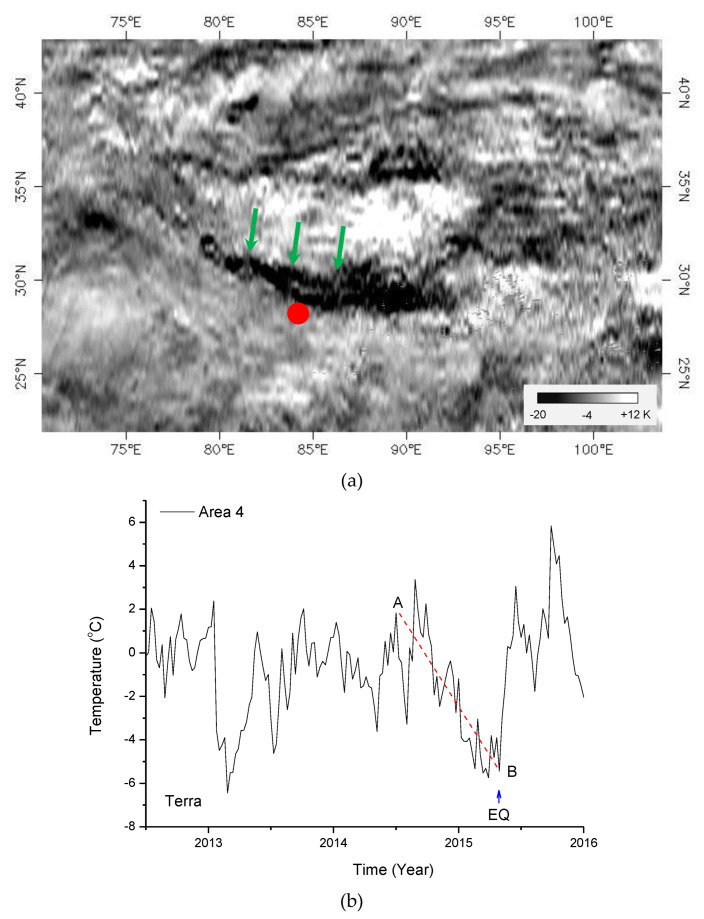
The case of the Terra satellite. (**a**) Spatial distribution of coseismic temperature (23–30 April 2015); (**b**) the temporal process of local temperature in study area 4. AB in panel (**b**) represents the cooling process before the earthquake, and EQ is the Nepal earthquake. The red solid cycle is the Nepal earthquake.

The results from the MODIS/Aqua satellite are given above. For comparative analysis, we also calculated the LST data from the MODIS/Terra satellite. The calculation process is consistent with that for the Aqua satellite, and the results are given in Figure 8. Figure 8a shows the spatial distribution of the coseismic response according to the MODIS/Terra satellite. As illustrated in Figure 8a, a clear coseismic cooling strip appears. Comparing Figure 6b with Figure 8a, the spatial distribution of the coseismic cooling zone for the Terra satellite is seen to be basically consistent with that for the Aqua satellite.

Among the above-mentioned results for the Aqua satellite, study area 4 is located in the main cooling zone, and Figure 8b also shows the change in local temperature over time in study area 4 before and after the Nepal earthquake. Figure 8b illustrates that before the earthquake, study area 4 showed continuous cooling (AB), which reached a low value near the time of the earthquake, and a rapid increase in temperature back to the normal background after the earthquake. Comparing Figure 7 with Figure 8b, the results for the two satellites Terra and Aqua are seen to be basically the same for the time course of study area 4.

In summary, for both the temporal process before and after the earthquake and the spatial distribution, the results obtained from the Terra satellite are basically consistent with those from the Aqua satellite. The data from different satellites yield the same results, which indicate that the cooling phenomenon is independent of satellites. In other words, the phenomenon is repeatable and reliable.

### 5.3. Physical Mechanism

There are several mechanisms that cause thermal infrared anomalies [23], mainly including deflation of gases such as CO2 and CH4 within the crust [9], p-hole excitation theory (Freund, 2007a, b), conversion between mechanical and thermal energy [22,44,45], and systematic coupling effects, such as seismic-atmosphere-ionosphere coupling [16,46]. Most physical mechanisms are associated with stress changes or their secondary effects, but each mechanism emphasizes different aspects of physical properties that are produced by or accompany stress changes, such as gas, electricity, magnetism or heat. In general, each physical mechanism itself has an experimental basis, and focuses on analysis of a conceptual model, which is helpful for establishing a qualitative understanding of satellite thermal infrared anomalies. 

The cooling phenomenon found in this paper was also found in another thrusting-type earthquake, namely the Wenchuan Mw7.9 earthquake, which occurred in Longmenshan thrust faults located at the Eastern boundary of the Qinghai-Tibet Plateau [27]. Additionally, there are some other cases of decreasing temperature anomalies [47]. Freund’s p-hole theory [18,19] is widely used as the physical basis of why a temperature rise can be observed, but for such a strong temperature drop as the ones reported by the present study and the other cases of decreasing temperature anomalies [27,47], there is not any physical basis explained, and this requires us to explore the other physical mechanism.

According to theoretical and experimental studies, temperature has a sensitive response to stress changes, and the temperature decreases when the extensional stress enhances [48,49,50,51]. So, there exists the possibility of the temperature drop as stress relaxation is generally accompanied by the occurrence of earthquakes. The Nepal earthquake was a thrust-faulting earthquake, and stress relaxation occurred on the upper side of the thrust fault (namely on the Northern side of the Nepal earthquake). The coseismic temperature decrease is qualitatively in accordance with the coseismic stress drop on the upper side of the thrust fault. As mentioned above, the values for the stress drops of the Nepal earthquake are highly dependent on the model [37]. Now, we cannot give more interpretation. Frankly, we cannot say that this explanation is final; however, we hope that it can provide some clues for approaching a reasonable physical interpretation in the future.

### 5.4. Implication of the Coseismic Temperature Response

Since the physical model is insufficient, if certain information from the process of thermal information change can be found, such information can be helpful to further understand the phenomenon. The following assumption seems to be plausible: for an earthquake-related signal, if there is an abnormality before the earthquake, the peak should be reached at the time of occurrence of this earthquake and should weaken after the earthquake. The factors that influence LST are extremely complicated; in particular, meteorological changes occur rapidly. Thermal information related to earthquakes might be overwhelmed by complex meteorological effects. In general, the coseismic response should be a relatively certain piece of information that may be used as a basis for determining whether other information is related to an earthquake.

Through the above analysis, during the Nepal earthquake, a cooling strip corresponding to the seismogenic structure was found. In particular, from the temporal perspective, the temperature slowly decreased before the earthquake, reached a minimum at the time of occurrence of the earthquake, and returned to a normal level after the earthquake. This sequence indicates that this spatial and temporal change is closely related to the earthquake and may have been caused by the tectonic activity associated with the Nepal earthquake. 

In addition, as mentioned above, the variation range of this signal was more than twice the maximum standard deviation among study areas (Figure 4). Meanwhile, there exists the physical possibility that the temperature drop with the stress relaxation is generally accompanied by the occurrence of an earthquake. Lastly, considering that these changes were related to and initiated before the earthquake, they might even have been potential precursors of the Nepal earthquake.

## 6. Conclusions

In this paper, a total of 15 or 17 years of MODIS/Aqua and MODIS/Terra satellite remote sensing LST products from 2002 or 2000 to 2016 was selected to analyze the temperature change before and after the Mw 7.9 earthquake in Nepal on 25 April 2015 and to explore possible thermal information related to earthquakes. A set of “physical” methods was used to analyze the effects of non-seismic factors such as solar radiation and atmospheric circulation. The results from the two satellites are listed below:

(1) From the time course, there was significant cooling before the earthquake in study areas 3 and 4, which reached the lowest values during the earthquake and returned to normal after the earthquake. The variation range was more than twice the maximum standard deviation among study areas, and even more than 4 times the maximum standard deviation among study areas. That is, in study areas 3 and 4, there occurred changes in temperature greater than normal backgrounds before the earthquake. This cooling peaked at the time of the earthquake and returned to normal after the earthquake. This sequence indicates that changes closely related to the earthquake over time may have been due to tectonic activity associated with the Nepal earthquake. Considering that these changes occurred before the earthquake, they may even have been precursors to the Nepal earthquake.

(2) From the spatial distribution, there were cooling strips corresponding to the seismogenic structure during the earthquake. These coseismic cooling strips were distributed along the Himalayas and were approximately 1300 km long. The widths of the East and West sides were slightly different, with an average temperature decrease of 5.6 °C. The Western section (AB) was approximately 90 km wide and approximately 500 km long; the East side (BC) was approximately 190 km wide and 800 km long. The Western side of the cooling strips appeared before the earthquake.

(3) From the relationship between the stress change and the temperature response, if possible, the above temperature change process might indicate that there was a progression in which the crustal stress gradually decreased before the earthquake, which is consistent with the coseismic stress drop of this thrust-type earthquake.

## Figures and Tables

**Table 1:** Statistics for local temperature $T_{local}\left(t,x,y\right).$

Study Area	Totals	Non–Smoothed				Smoothed			
		Mean	Std ^a^	Max	Min	Mean	Std	Max	Min
No. 1	641	0.11	1.42	3.91	−4.51	0.11	0.64	2.02	−1.66
No. 2	641	−0.03	1.39	3.71	−4.35	−0.02	0.62	1.57	−1.63
No. 3	641	−0.17	1.55	4.61	−4.66	−0.17	0.75	1.37	−2.86
No. 4	641	−0.20	1.65	3.64	−5.83	−0.20	0.95	1.37	−4.60
No. 5	641	0.11	1.42	3.91	−4.51	0.04	0.37	1.27	−1.28

^a^: Standard deviation of time-series data; Note: the unit of local temperature is °C.

**Figure A1:**
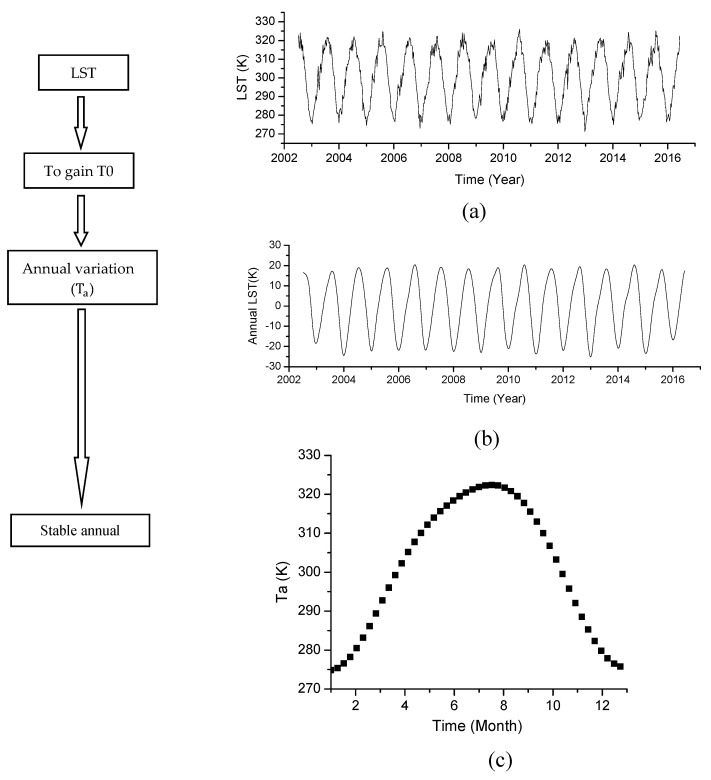
The case of obtaining the stable annual variation ($T_{sta}$). (**a**) Land surface temperature at a fixed position; (**b**) annual variation in LST with annual periodicities in the range of 256–512 days; (**c**) stable annual variation field.

**Figure A2:**
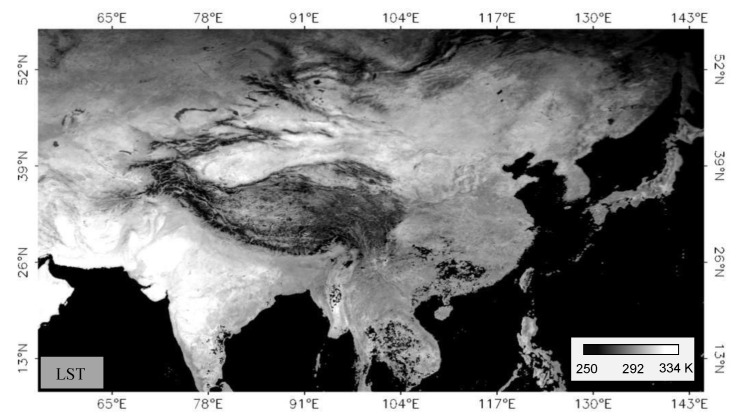
Land surface temperature at the time of the Nepal earthquake (Aqua) (23–30 April 2015).

**Figure A3:**
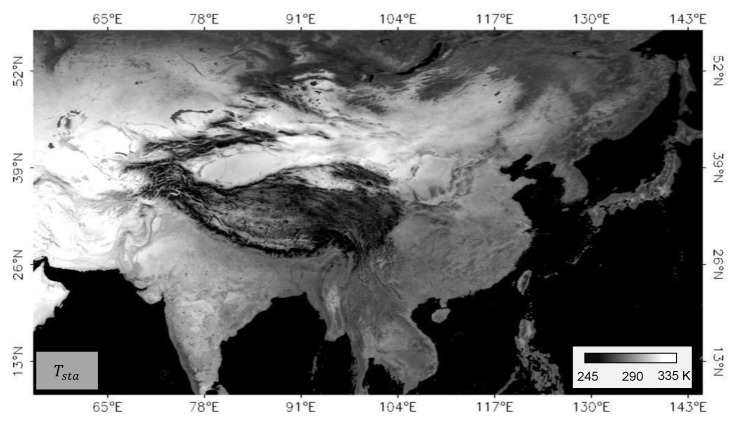
Spatial distribution of $\;T_{sta}$ (23–30 April 2015).

**Figure A4:**
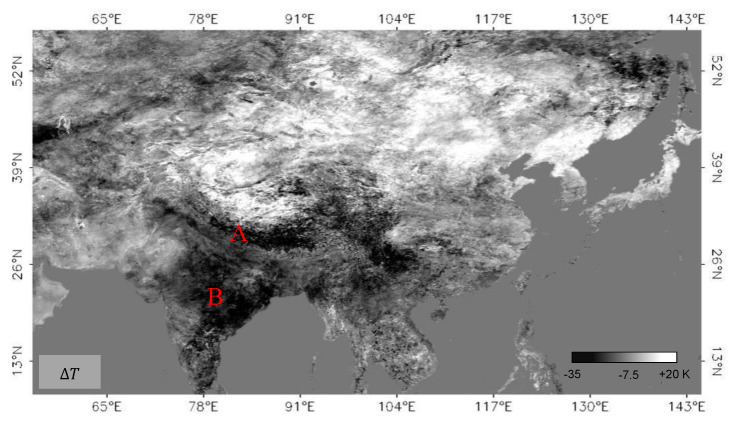
Spatial distribution of ΔT (23–30 April 2015).

**Figure A5:**
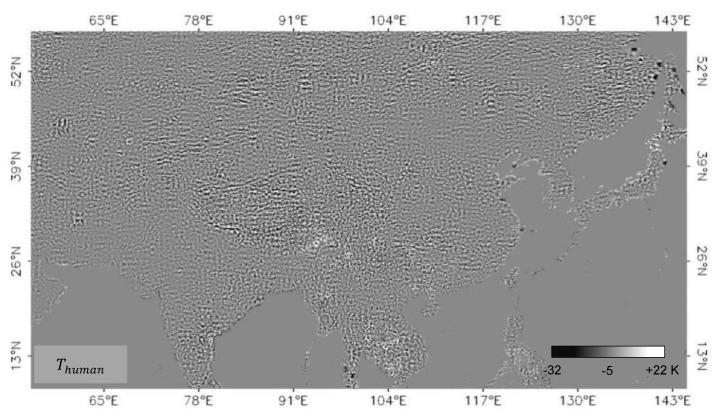
Spatial distribution of $T_{human}$. Spatial scales: less than 80 km × 80 km.

**Figure A6:**
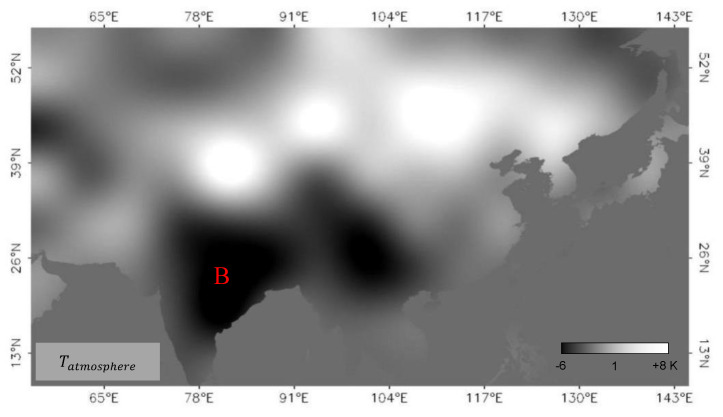
Spatial distribution of $T_{atmosphere}$. Spatial scales: greater than 600 km × 600 km.

**Figure A7:**
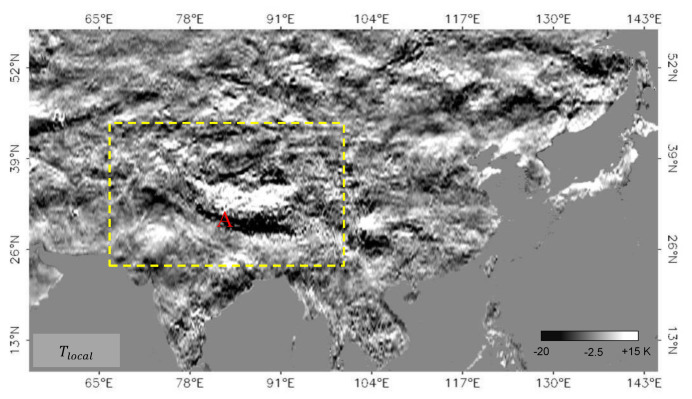
Spatial distribution of $T_{local}$**.** Spatial scales: greater than 80 km × 80 km and less than 600 km × 600 km. Note: the rectangular box in $T_{local}\;$ is the research range given in the main text (namely, Figure 1a).

## Appendix A. One-Dimensional Wavelet Analysis

Wavelet transformation is a theory that has been widely applied in mathematics and other fields of science and technology since the end of the 20th century [52,53]. The best feature is the auto-adaptability and mathematical microscope. This theory can automatically adjust relevant parameters according to the object being studied and elucidate time-frequency local properties. Wavelet analysis is derived from traditional Fourier analysis but avoids the limitation that Fourier analysis cannot yield local information, and wavelet analysis can provide spectral characteristics of signals over time [54].

For a given wavelet function h(x), a family of functions is obtained by expansion and translation [52]:

(A1) $$h_{j,k}(x)=2^{j/2}h(2^{j}x-k)$$

where *j* is the scaling factor and k is the translation factor. The smaller the scale is, the higher the frequency; the larger the scale is, the lower the frequency. The wavelet transform at any scale is:

(A2) $$C(j,k)=<h_{j,k},f>=2^{j/2}\int \bar{h_{j,k}(x)}f(x)dx$$

where C(j,k) is the wavelet coefficient and $\underline{h_{j,k}\left(x\right)}$ is the conjugate of $\;h_{j,k}\left(x\right)$.

For the square integral function space $\;L^{2}\left(R\right)$, any given signal $f\left(x\right)\in L^{2}\left(R\right)$ can be decomposed into different frequency bands by scale [52,53]:

(A3) $$f(x)=\sum_{j,k=-\infty }^{\infty }C(j,k)h_{j,k}(x)$$

(A4) $$\;=\cdots +g_{-1}\left(x\right)+g_{0}\left(x\right)+g_{1}\left(x\right)+\cdots +g_{j}\left(x\right)+\cdots \;$$

where $g_{j}\left(x\right)$ is the component of f(x)  on scale *j*.

If h is an orthogonal wavelet, the components in (A4) are orthogonal to each other, namely:

(A5) $$<g_{i}(x),g_{j}(x)>=0(i\neq j)\;$$

where $<g_{i}\left(x\right),\;g_{j}\left(x\right)>$ represents the inner product of the two components, and if the inner product is zero, the components are uncorrelated and can be added in any combination without being affected by other components. Selecting the appropriate frequency band $g_{j}\left(x\right)\;$ can be accomplished by combining Equation (A4) with Equation (1).

In theory, Equation (A4) can be infinitely decomposed, but in fact, j cannot be too large; only a limited number of wavelet coefficients can be processed. To accurately calculate these coefficients, the size of j should be appropriate [54]. In this paper, the orthogonal wavelet Coiflet is used for decomposition. The appropriate decomposition number corresponding to the wavelet Coiflet is 5, which is the number of decomposition layers in this paper.

## Appendix B. Two-Dimensional (2D) Wavelet Analysis

Two-dimensional (2D) analysis is a common problem in signal processing, such as image processing. Two-dimensional wavelet analysis is one of the important methods of two-dimensional signal processing [42].

Assuming a signal function $\;f\left(x,y\right)\in L^{2}\left(R^{2}\right)$, φ(x,y)  is a two-dimensional mother wavelet function whose construction can be formed by the tensor product of the one-dimensional mother wavelet or by the nontensor product method, $\varphi_{a,b,c}\left(x,y\right)={\left|a\right|}^{2}\varphi \left(\frac{x-b}{a},\frac{x-c}{a}\right),b,c\in R,\;a\in R-\left\{0\right\}$.

The two-dimensional continuous wavelet transform is:

(A6) $$Wf(a,b,c)=CWT(a,b,c)=|a{|}^{-1}\iint f(x,y)\varphi (\frac{x-b}{a},\frac{y-c}{a})dxdy$$

If we set $\;a=2^{-j}$,$\;b=k_{1}2^{-j}$, and $\;b=k_{2}2^{-j}$,$\;j,k_{1},\;k_{2}\in Z$, the two-dimensional discrete wavelet transform is:

(A7) $$DWT(j,k_{1},k_{2})=2^{j}\sum_{l_{2}}\sum_{l_{1}}f(l_{1},l_{2})\varphi (2^{j}l_{1}-k_{1},2^{j}l_{2}-k_{2})$$

where $\;l_{1},l_{2}\in Z$.

Assume that the dual wavelet corresponding to the two-dimensional mother wavelet φ(x,y)  is $\underline{\varphi }\left(x,y\right),\;{\underline{\varphi }}_{a,b,c}\left(x,y\right)={\left|a\right|}^{2}\underline{\varphi }\left(\frac{x-b}{a},\frac{x-c}{a}\right)$. Then, there is a unified reconstruction equation corresponding to the two-dimensional wavelet transform, namely, the inverse wavelet transform:

(A8) $$f(x,y)=<f(x,y),\varphi_{a,b,c}>{\bar{\varphi }}_{a,b,c}=<f(x,y),{\bar{\varphi }}_{a,b,c}>\varphi_{a,b,c}$$

where <, > represents the inner product of the two functions. The discrete case represents the sum of the product of the corresponding components.

According to the two-dimensional multiresolution analysis theory and the Mallat algorithm (Mallat, 1989), a two-dimensional function  f(x,y), after a two-dimensional wavelet decomposition, can be decomposed into a sum of a smooth component and three detail components. After being decomposed by the J layer, there is the following:

(A9) $$f(x,y)=A_{-J}f+\sum_{j=-1}^{-J}\sum_{e=1}^{3}D_{j}^{e}f$$

where *J* is a positive integer, $A_{-J}f$ represents the smoothed component after the *J*-layer decomposition and $D_{j}^{e}f$ represents the *e*-th detail component of the *j*-th layer decomposition. The above equation is a multiresolution decomposition equation for two-dimensional wavelets, namely, the Mallat algorithm [43].

In Equation (A9), the various components obtained by the decomposition of the J layer are essentially the decomposition of the time-frequency space. If the orthogonal wavelet is used to complete the above decomposition, the components are orthogonal to each other, and the signals are independent of each other. According to the previous analysis, there is a significant spatial scale difference between the atmospheric circulation and the in situ temperature in Equation (3). That is, Equations (3) and (A9) are similar. The wavelet multiresolution analysis represented by Equation (A9) can separate the atmospheric circulation and the in situ temperature components in the annual variation. From the LST field, the in situ temperature component generated by the local factor is extracted, and information on crustal activity can be found. This process is also a new application for wavelet analysis.

## Appendix C. The Stable Annual Variation ($T_{sta}$) in Land Surface Temperature (LST)

Since both T0 and Ta are stable in LST, they can be merged as a stable annual variation field (SAVF), Ta. SAVF is the main component of LST and can be taken as the background because solar radiation is the factor with the strongest influence (Figure A1).

SAVF can be obtained from the LST product by the following steps [55]: (1) Remove the influence of non-annual components with the help of a curtain method for filter waves. Meanwhile, the annual signal should be retrieved. Wavelet analysis is applied to retrieve the annual component in order to reduce the influence of other periodic signals and errors produced by filtering, with annual periodicities in the range of 256–512 days. One of the advantages of the orthogonal wavelet method is that the retrieved annual periodic components barely experience interference by other components [42,43]. (2) Obtain SAVF by averaging the annual temperature signals for the same calendar days. As shown in Figure A1, SAVF is similarly sinusoidal, which agrees with the solar radiation during Earth’s revolution.

## Appendix D. Spatial Distribution of Land Surface Temperature and Its Components

It can be seen from the procedure of LST processing that $T_{local}\left(t,x,y\right)\;$ is obtained from the LST and involves various temperature data, including $T_{a}\left(t,x,y\right)$, ΔT(t,x,y), $T_{atmosphere}\left(t,x,y\right)$, $T_{local}\left(t,x,y\right)$ and $\;T_{human}\left(t,x,y\right)$. This article mainly analyzes $\;T_{local}\left(t,x,y\right)$ and does not involve other components. Here, we present a case for the spatial distribution of LST and its components.

To obtain more stable results, the data range selected in this paper is larger than the scope of the text. Here, we take the spatial results from the Aqua satellite as an example and give the spatial distributions of LST and its various temperature components (Figure A2, Figure A3, Figure A4, Figure A5, Figure A6 and Figure A7). As mentioned in Section 3.3, in order to avoid the border effect on the spatial decomposition, the spatial range is larger than those of Figure 2a, as shown in Figure A2, Figure A3, Figure A4, Figure A5, Figure A6 and Figure A7.

Figure A2 shows the LST at the occurrence of the Nepal earthquake. From the LST field, it is difficult to distinguish information related to earthquakes. Contrasting $\;T_{sta}\left(t,x,y\right)$ and  T(t,x,y) shows that the spatial distribution of the two is largely similar (Figure A2 and Figure A3). The analysis of the time process illustrates that $\;T_{sta}\left(t,x,y\right)$ is a stable annual cycle component that is independent of crustal activity and a stable static component that has the strongest influence on LST.

In ΔT(t,x,y), a temperature decrease (A) can be seen near the epicenter area (Figure A4). As shown in Figure A4, there are still other obvious cooling zones (B). Further, ΔT(t,x,y) is decomposed into $\;T_{atmosphere}\left(t,x,y\right)$, $T_{local}\left(t,x,y\right)$ and $\;T_{human}\left(t,x,y\right)$, as shown in Figure A5, Figure A6 and Figure A7. The cooling zone B in Figure A4 is moved into $\;T_{atmosphere}\left(t,x,y\right)$ because it is more likely to be an atmospheric circulation effect. At the same time, in the spatial distribution of $\;T_{local}\left(t,x,y\right)$. the cooling phenomenon (A) near the epicenter is much clearer (Figure A7).

Note that the rectangular box in the spatial distribution of $T_{local}\left(t,x,y\right)\;$ in Figure A7 is the research range given in the main text (Figure 1a).
